# Ensembles in solution as a new paradigm for antibody structure prediction and design

**DOI:** 10.1080/19420862.2021.1923122

**Published:** 2021-05-25

**Authors:** Monica L. Fernández-Quintero, Guy Georges, Janos M. Varga, Klaus R. Liedl

**Affiliations:** a Department of General, Inorganic and Theoretical Chemistry, and Center for Molecular Biosciences Innsbruck (CMBI), University of Innsbruck, Innsbruck, Austria; b Roche Pharma Research and Early Development, Large Molecule Research, Roche Innovation Center Munich, Penzberg, Germany

**Keywords:** Antibody structure, antibody structure prediction, antibody design, ensembles in solution

## Abstract

The rise of antibodies as a promising and rapidly growing class of biotherapeutic proteins has motivated numerous studies to characterize and understand antibody structures. In the past decades, the number of antibody crystal structures increased substantially, which revolutionized the atomistic understanding of antibody functions. Even though numerous static structures are known, various biophysical properties of antibodies (i.e., specificity, hydrophobicity and stability) are governed by their dynamic character. Additionally, the importance of high-quality structures in structure–function relationship studies has substantially increased. These structure–function relationship studies have also created a demand for precise homology models of antibody structures, which allow rational antibody design and engineering when no crystal structure is available. Here, we discuss various aspects and challenges in antibody design and extend the paradigm of describing antibodies with only a single static structure to characterizing them as dynamic ensembles in solution.

## Introduction

Antibodies are protective agents used by the adaptive immune system to recognize and neutralize foreign objects through interactions with the target antigen. Long half-life, specificity to their respective antigen, and efficacy are beneficial attributes of antibodies.^1^ Because of their ability to recognize targets, they offer an innovative and efficient way to control pathogens by binding to their surfaces and thereby inactivating them. The immunoglobulin repertoire contains enormous diversity, which facilitates the recognition of a wide variety of different antigens. Antibodies have become one of the fastest growing fields in terms of academic and industrial research.^1^ Three of the top 5 selling drugs in 2019, 2020 and 2021 are in fact antibodies.^2–4^ This substantial interest has led to a vast amount of experimental data, including affinity and stability measurements as well as structural information.

## The antigen binding fragment

The ability of an antibody to recognize a broad variety of different pathogens, such as viruses and bacteria, is determined by the antigen-binding fragment (Fab). This region consists of a heavy and a light chain that can each be subdivided into a constant (C_H_1, C_L_) and a variable domain (V_H_, V_L_).^5^ The variable region, also known as Fv, is the focal point of recombination and somatic hypermutation events.^6–8^ The diversity of an antibody in sequence and structure is concentrated within six hypervariable loops, the so-called complementarity-determining regions (CDRs), forming the antigen-binding site of an antibody.^9–12^ The heavy and light chains contain three loops each, known as CDR-H1, CDR-H2, CDR-H3 and CDR-L1, CDR-L2, CDR-L3, respectively.

Although there is great variation in the sequence and size of the CDRs, five of the six loops (CDR-H3 is the exception) have been classified into so-called canonical structures, assuming that they can only adopt a limited number of backbone conformations.^11,13–18^ Furthermore, the different amino acids at position H71 (Kabat nomenclature)^10,12^ are thought to influence both the position and the canonical cluster assignment of the CDR-H2 loop, and thus potentially affect antigen binding.^17,19,20^ Generally, the major determinants of specificity and affinity of these five CDR loops for an antigen are the size, shape and biophysical complementarity of their surface residues and their relative positions to each other.^11^ The CDR-H3 loop reveals the highest diversity in length, sequence and structure and has the ability to adopt various different conformations during the V(D)J recombination and somatic hypermutation. Thus, the accurate prediction of CDR-H3 loop structure remains challenging.^21–23^ The CDR-H3 loop is also known to play a central role in antigen-binding and recognition as it has on average the highest counts of contacts with the antigen.^21^ Additionally, the length and structure of the CDR-H3 loop can directly influence the antigen-binding patterns, and thereby have an effect on the specificity of the paratope.^21,22^

Recent studies that investigated the conformational diversity of the CDR-H3 loop in solution have shown that, in particular, CDR-H3 loop conformations in unbound antibody X-ray structures can be distorted by crystal packing effects and that the actual dominant CDR-H3 loop conformation in solution is optimized to bind the antigen. Thus, special care has to be taken when characterizing antibody CDR-H3 loops based on “unbound” Fab X-ray structures.^24^

Furthermore, it was shown that one single static structure is not enough to capture the high flexibility of any of the CDR loops. All CDR loops, not just CDR-H3, should thus be described as conformational ensembles in solution. Conformational rearrangements of the individual CDR loops and transitions between different canonical clusters were observed in the micro-to-millisecond timescale. Some canonical clusters even belong to the same kinetic minimum in solution, and hence might be combined.^25,26^

The regions of the variable domains apart from these loops are known as framework and are highly conserved in both sequence and main-chain conformations.^10–12^ This variability in the antigen-binding site is achieved by V(D)J recombination,^27^ somatic hypermutation,^6^ class switching^7^, and the combinatorial diversity via heavy and light-chain pairing.^9^ Apart from the length and sequence composition of the CDR loops, the relative orientation of V_H_ and V_L_ codetermines the shape of the antigen-binding site. Reorientations in the relative V_H_-V_L_ orientation directly change the binding site geometry, and thereby have an effect on the specificity and affinity of the paratope. Especially in the field of antibody engineering, the preservation of the V_H_-V_L_ orientation is essential to retain the original antibody properties.^28–30^ The V_H_-V_L_ interface also strongly influences the stability of the Fv region. Because numerous residues in the V_H_-V_L_ binding interface are highly retained, the role of conserved residues on the Fab function and consequently binding has been studied. Mutations that are distant from the CDR loops, however, also have effects on binding, which indicates that they indirectly affect antigen binding by favoring different V_H_-V_L_ interface orientations.^31–33^ In addition, the influence of amino acids at position H23 (Kabat nomenclature)^34^ have been shown to have an effect on antigen-binding.^33^ Changes in the V_H_-V_L_ interdomain orientations of up to 5° have also been reported upon antigen-binding and have been interpreted to follow the induced-fit mechanism of antigen recognition through rigid-body rotations of the V_H_ and V_L_ domains.^35,36^

Molecular dynamics simulations of whole Fvs and Fabs reveal fluctuations in these relative V_H_-V_L_ interdomain orientations.^37^ The observed variability between these domains has been confirmed by nuclear magnetic resonance (NMR) experiments and is in line with the idea that these relative interdomain orientations can be interpreted as an additional structural feature of antibodies that increases the antibody repertoire and enlarges the number of possible binding partners. By applying fast Fourier transformation to the interface angles, timescales of 0.1 to 10 GHz could be assigned to the fastest collective interdomain movements, while the slower components of the observed dynamics are governed by conformational changes in the CDR loops that occur in the micro-to-millisecond timescale.^37,38^

**Figure 1.**
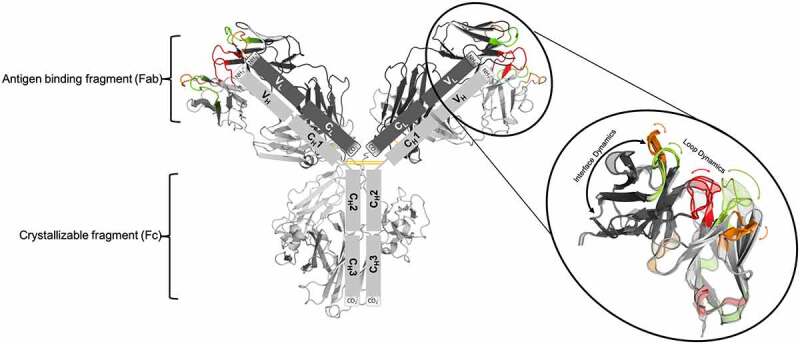
Structure of an IgG1 antibody and schematic illustration of the unique modular anatomy. the arms of the Y-shaped structure allow the antibody to carry out two functions, on the one hand antigen-binding and on the other hand biological activity mediation. the arms of the antibody are known as antigen-binding fragments (Fabs). The Fab is composed of a constant and a variable domain of each of the heavy and the light chain. the variable domains shape the antigen binding site (paratope) at the amino-terminal end of the antibody. the variable fragment (Fv) is highlighted in the picture. the CDR 1 loops are colored in green, the CDR 2 loops are depicted in orange and the CDR 3 loops are shown in red. The close up to the Fv also indicates the high flexibility of the CDR loops and the relative V_H_-V_L_ interface and shows that the antibody binding site exists as ensembles of paratope states. the tail region of the antibody, also known as Fc region, is responsible for the communication with the immune system and interacts with the cell surface receptors, called Fc receptors

**Figure 2.**
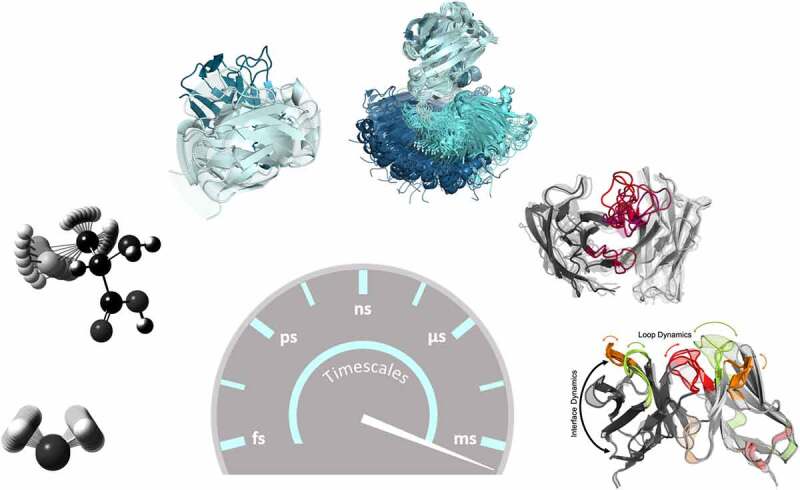
Antibodies exist as ensembles in solution. summary of antibody Fab dynamics and their respective timescales. bond vibrations and sidechain rotations can already be captured in the femto-to-picosecond timescale. Interface and elbow angle dynamics occur in the low nanosecond timescale, while conformational transitions between CDR loops can be sampled in the microsecond timescale. the combination of interface angles and different CDR loop conformations have been described as ensembles of paratope states in solution, which interconvert between each other in the micro-to-millisecond timescale

In contrast to the prevalent static view of the binding interface, it was shown that antibodies exist as ensembles of paratope states.^39^ These paratope states are defined by a characteristic combination of CDR loop conformations and interdomain orientations. They interconvert into each other in the micro-to-millisecond timescale by correlated loop and interdomain rearrangements. Several studies have shown that crystal packing effects in unbound crystal structures can distort the paratope and thus result in misleading X-ray structures.^24,40^ For the first time, a complete description of conformations, thermodynamics and kinetics of the whole-binding paratope in solution can be achieved, which provides a new paradigm in the understanding of CDR binding loop states, antibody-antigen recognition, relative V_H_-V_L_ interface and elbow angle distributions and their respective dynamics (Figures 1 and 2). In addition, it has been shown that these conformational ensembles also determine the hydrophobicity of antibodies, which makes them particularly relevant for tackling antibody developability issues.^41,42^

The overall stability of a Fab is governed by the high degree of cooperation between the elbow angle and the V_H_/V_L_ and C_H_1/C_L_ interface, while the direct interactions of the V_L_ and C_L_/V_H_ and C_H_1 domains do not influence the stability of either domain.^43^ Similar to the relative V_H_-V_L_ interface, the C_H_1-C_L_ interdomain orientations also reveal high variability and can be captured in the low nanosecond timescale. However, even though the captured dynamics are similar between the Fab interfaces, the nature and number of interface interactions can differ. The constant domains of the Fab show hydrophobic interactions at the center of the interface surrounded by a small number of salt-bridges, while the Fv interface is strongly dominated by framework interactions and conformations of the CDR loops.^34,43^ Structurally, the C_H_1-C_L_ domains resemble the C_H_3-C_H_3 domains. Apart from the V_H_/V_L_ and C_H_1/C_L_ interface, the elbow angle is also influenced by the shape of the paratope and might contribute to antigen specificity. The elbow angle is defined as the angle between the pseudo-2-fold axes relating to V_H_-V_L_ and C_H_1-C_L_, and has been shown to increase Fab flexibility and allow the same antibody to recognize different antigens.^44,45^ Mutations in the Fab elbow region have been reported to influence conformational flexibility and paratope plasticity.^19,45–49^

## The crystallizable fragment region

The tail region of the antibody, known as the crystallizable fragment (Fc), is responsible for interactions with the cell surface, immune system activation and extension of the molecular half-life.^5^ Antibody Fabs and Fc domains are linked together via a flexible unstructured hinge region. The Fc can be divided into a C_H_2-C_H_2 and a C_H_3-C_H_3 dimer. The C_H_2-C_H_2 dimer is mainly responsible for interacting with type I or type II Fc receptors (FcRs), which can be located on effector cells or on B cells, and thereby modulate both the adaptive and innate immune response. The interface between the two C_H_2-C_H_2 domains contains conserved glycosylation sites at Asn297, which are conjugated to a core heptasaccharide, forming a biantennary Fc glycan. The glycans modulate the functions, affinities and Fc conformations.^50–53^ The hydrogen bonding in the C_H_2-C_H_2 interface can be observed either directly between the two carbohydrate chains, or through a dynamic water network.^54^ Detailed structural and dynamic analysis of the C_H_2-C_H_2 interface in IgG1 and IgG2 has revealed that movements of the C_H_2 domains originate from pivoting around a highly conserved ball- and socket-like joint, formed by the C_H_2 L251 sidechain (ball) with the C_H_3 residues M428, H429, E430 and H435 (socket).^54^

The C_H_3 domains bind tightly with each other by both hydrophobic interactions at the center, surrounded by salt bridges, thereby forming the foundation for the heavy-chain dimer association.^54^ Mutations in the C_H_3-C_H_3 interface have been shown to not only strongly influence the stability and the association of the two domains, but also alter glycosylation and result in structural changes of the C_H_2 domain.^55^ By mutating residues in the interface, the energetic contributions of single amino acids could be quantified. Thereby, three contacts within the interface were found to highly stabilize the interface, with the hydrogen bond between T366 and Y407 in the center of the interface described as the most important interaction. Similarly, the charge–charge interaction between K409 and D399 was shown to have a high energetic contribution, as well as the hydrophobic interactions of L368 and F405.^56,57^

Heterodimeric Fc variants have been engineered primarily through the replacement of homodimer-favoring interactions at the interface with heterodimer-favoring interactions by asymmetric mutations in both heavy chains. These rational approaches can be classified into different strategies, with some of the strategies relying on steric complementarity (also known as the Knobs-into-Holes approach), and others involving the introduction of asymmetric charged interactions.^58,59^

Various studies have investigated the influence of the Fab, the Fc and the glycans on the activity of an antibody.^52,60^ It was recently shown that antigen binding induces conformational changes in the Fc domain, followed by Fc receptor activation. Thus, antigen binding also allosterically promotes Fc receptor binding and recognition.^61^ Consequently, conformational rearrangements in the Fc directly modulate the activity and binding affinity toward binding and recognizing Fc receptors.^52^

## Antibody specificity – antibody affinity maturation

The most striking aspect of antibodies, and at the same time a fundamental requirement of the immune system, is the specific nature of their interaction with an antigen.^62^ The specificity of an antibody evolves through various rounds of somatic hypermutations, followed by selection in the germinal centers.^6,63^ Repeated exposure of the same antigen results in a selection of antibodies with higher affinities and specificities. Studies investigating various different aspects of humoral and cellular immunity have contributed to the present view of specificity as part of the complexity of molecular recognition.^7,64–68^

Antibodies were first identified at the end of the 1800s, yet the process by which can a limited repertoire of antibodies recognize an effectively limitless number of antigens is still not fully understood.^69^ Sufficient evidence showing that antibodies are not infinitely specific has accumulated. Numerous studies have in fact demonstrated that antibodies can recognize more than one antigen and thus can be described as functionally promiscuous or multi-specific.^66,69–72^ This was already discussed in the 1940s, when Pauling and Landsteiner suggested that antibodies follow the concept of conformational diversity.^73,74^

**Figure 3.**
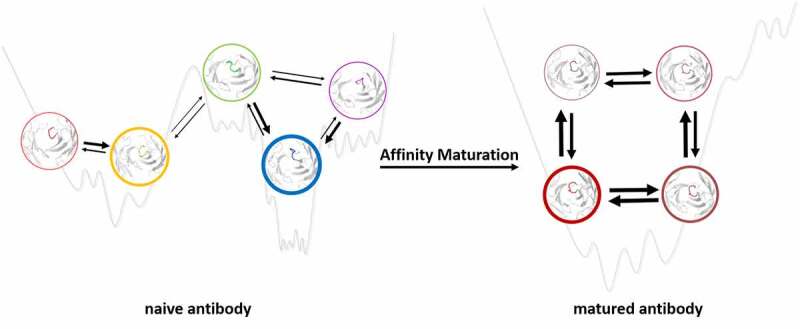
Effect of maturation on the free energy landscape. the potential energy hypersurface of the naive and the matured antibody are represented as 1D basins, showing accessible conformational substates. the wide basins of the naive antibody illustrate the possibility of binding a diverse set of antigens. The increased depth and at the same time decreased number of basins upon affinity maturation indicate the enhanced enthalpic interactions that formed, which are accompanied by a decrease in conformational entropy

Following Landsteiner’s idea that there are ‘different ways of folding the same polypeptide chain’, Pauling proposed the idea of having an ensemble of preexisting conformations out of which the functional ones are selected.^73^ This view was also supported by the conformational selection or population shift model originating from the Monod-Wyman-Changeux model.^75–77^ In the early 1990s, Milstein and Foote revived this idea,^78,79^ which was subsequently also demonstrated Wedemayer.^80^ The concept of conformational selection suggests that, within this preexisting ensemble of conformations, the binding competent state is selected, accompanied by a population shift.^76,81^ The probability of the conformation chosen by the antigen determines the binding mechanism, which can be either “lock and key”,^36,82^ “conformational selection”,^75,76,83^ or “induced fit”.^36,83,84^ Historically, protein–protein interactions such as antibody-antigen binding were assumed to follow the “lock and key” mechanism. This “lock and key” binding mechanism can especially be observed for matured antibodies, where the apo conformation is selected as the binding competent conformation.^80,85^ Studies investigating the consequences of affinity maturation have observed a substantial rigidification of the antigen-binding site as a consequence of the increase in specificity.^38,39,80,85–87^ Even though rigidification might only be one of the various consequences of affinity maturation, it still represents a fundamental mechanism resulting in an increase in specificity (Figure 3).

If the binding occurs to a rare conformation in solution, which cannot be detected before binding, the process can be interpreted as induced fit binding.^88^ Both induced fit and conformational selection have been discussed in current literature to elucidate the binding preferences of multi-specific antibodies, which can recognize various structurally unrelated antigens with low affinity due to their inherently more flexible-binding site.^65,72,83,84^ Thus, promiscuity might arise from a multitude of weakly populated conformations, each of which is able to bind different binding partners. Rigidification upon affinity maturation shifts the probabilities toward a smaller number of states, and thereby reduces the number of potential-binding partners.^86^

## Future perspective and recommendations to the community

As the functions and properties of antibodies are strongly governed by their dynamic nature, both Fab as well as Fcs should be considered as ensembles in solution. Especially, the Fab, which is responsible for antigen binding and recognition, should be described as having interconverting states in solution. The probabilities of these states determine the specificity, promiscuity and affinity. These different conformations of the antigen-binding site are characterized by different paratope states in solution and CDR loop state-dependent interdomain orientations. In-depth understanding of these states and their dynamic interconversion is a paradigm change for rational antibody design and engineering. Furthermore, allosteric effects resulting in signal transduction from the antigen-binding site, reaching as far as to the Fc receptor-binding site, have to be expected. This signal is surmised to be transmitted by interdomain rearrangements of the V_H_-V_L_, C_H_1-C_L_, C_H_2-C_H_2 and C_H_3-C_H_3 interfaces.

Thus, what could be done differently in practice? First of all, the one single structure characterizing an antibody the best is the dominant conformation in solution, which does not necessarily coincide with the (apo) X-ray structure. The community should strive to predict this dominant structure in solution instead of trying to predict X-ray structures potentially distorted by crystal packing effects. Obviously, developing such predictions is a time- and resource-consuming effort, as it is necessary to systematically characterize and, if possible, experimentally verify (e.g., by NMR), a large number of dominant conformations in solution. For a deeper understanding of binding properties (e.g., finetuning of specificity) and eventually also other biophysical properties (e.g., developability liabilities), only looking at the dominant structure in solution is not sufficient. These properties can only be understood quantitatively by considering all important structures in solution weighted by their probabilities. In particular, docking might profit from such an approach.

## List of Abbreviations

CDRs – complementarity-determining regions

Fab – antigen-binding fragment

Fc – crystallizable fragment

Fv – variable region

NMR – nuclear magnetic resonance
